# Engagement modes and attitude polarization toward AI: the role of cognitive load and reliability among Chinese undergraduates

**DOI:** 10.3389/fpsyg.2025.1596330

**Published:** 2025-08-01

**Authors:** Duan Bo, Aini Azeqa Ma’rof, Zeinab Zaremohzzabieh, Li Rongfeng, Zheng Danhe

**Affiliations:** ^1^Faculty of Human Ecology, Universiti Putra Malaysia, Serdang, Malaysia; ^2^Department of Management, Shanxi Vocational University of Engineering Science and Technology, Taiyuan, China; ^3^Institute for Social Science Studies, Universiti Putra Malaysia, Serdang, Malaysia; ^4^Women and Family Studies Research Center, University of Religions and Denominations, Qom, Iran; ^5^Department of Music and Dance, Jingdezhen University, Jingdezhen, China

**Keywords:** artificial intelligence, attitude polarization, cognitive load, perceived reliability, information engagement, social psychology

## Abstract

**Introduction:**

This experimental study investigates how engagement modes with AI-related information—structured courses, group discussions, and self-directed research—influence attitude polarization and policy preferences among 132 Chinese undergraduates at a northern Chinese university. Methods: Participants were randomly assigned to conditions over a six-week intervention, with cognitive load and perceived reliability assessed as key mechanisms.

**Methods:**

Participants were randomly assigned to conditions over a six-week intervention, with cognitive load and perceived reliability assessed as key mechanisms.

**Results:**

Hierarchical regression revealed structured courses, marked by high cognitive load and reliability, significantly reduced polarization (*β* = −0.32, *p* < 0.01, η^2^ = 0.11), while self-directed research increased it (*β* = 0.45, *p* < 0.01, η^2^ = 0.15). Self-reported polarization strongly correlated with pre-to-post-test shifts (*r* = 0.68, *p* < 0.001), validating the General Attitudes Toward Artificial Intelligence Scale (GAAIS). Policy preferences mirrored these shifts, with structured courses fostering balanced stances (mean change = −0.15, SD = 0.40, *p* < 0.05).

**Discussion:**

This study suggests structured, reliable, cognitively demanding interventions mitigate polarization, offering theoretical insights into attitude formation and practical guidance for AI education and policy design.

## 1 Introduction

AI technologies have rapidly evolved, catalyzing societal discourse on their transformative potential and ethical dilemmas, as noted in prior research (Helbing, 2019). As AI penetrates domains like healthcare, education, and finance, it delivers tangible benefits—enhanced operational efficiency, personalized service delivery, and innovative breakthroughs. Yet, these advancements simultaneously evoke substantial concerns regarding privacy breaches, fairness in algorithmic decision-making, and the erosion of human oversight, necessitating a rigorous ethical framework to guide their deployment (Fast and Horvitz, 2017). The inherent complexity of AI systems, demanding significant cognitive effort for comprehension, further complicates public engagement and perception formation. Given AI’s pervasive reshaping of daily life, understanding public attitudes toward this technology is paramount for ensuring its ethical integration and ameliorating associated risks.

Public attitudes toward AI are markedly polarized, reflecting a spectrum of perspectives across demographic, socio-political, and cultural divides (Eurobarometer, 2021). Proponents extol AI’s capacity to boost productivity and elevate societal well-being, whereas skeptics caution against risks such as workforce displacement, privacy violations, and unintended societal consequences (Kellner, 2021). Quantitative evidence substantiates this divide: the Eurobarometer survey (Eurobarometer, 2021) reported that 57% of Europeans view AI favorably, while 36% express reservations about its implications.

Similarly, Zhang and Dafoe (2020) found that 41% of Americans support AI development, 22% oppose it, and 37% remain undecided. This fragmented sentiment highlights an urgent need to explore the underlying drivers of polarization, encompassing not only social influences but also the cognitive processes through which individuals interpret AI-related information. At the core of this attitudinal divergence lies the mode and nature of information exposure in the digital era. Individuals encounter AI-related content through diverse channels—mainstream news outlets, social media platforms, academic exchanges, and informal peer interactions—each presenting competing narratives about AI’s capabilities and risks (Bozkurt et al., 2023). These engagement modes vary in structure and cognitive demands, influencing the depth and direction of attitude formation.

Research indicates that passive engagement, such as news consumption, contrasts with active participation, like discussions, in shaping attitudes, often amplifying polarization when information is biased or contradictory (Tucker et al., 2018). Social psychology offers critical insights Festinger’s (1954) social comparison theory (SCT) posits that individuals refine their beliefs through interpersonal comparisons, potentially fostering extreme positions in uncertain domains like AI. Likewise, group polarization theory (Isenberg, 1986) suggests collective discussions intensify initial attitudes, an effect possibly magnified by varying perceptions of AI’s reliability and societal impact. These social dynamics intersect with cognitive effort, shaping how attitudes emerge and solidify.

This study employs a randomized experimental design, assigning 132 undergraduate students from a northern Chinese university to one of three engagement modes—structured courses, group discussions, or self-directed research—over a six-week intervention. By examining the interplay of cognitive load, perceived reliability, and exposure frequency, it seeks to clarify their influence on attitude polarization toward AI and related policy preferences. The research aims to identify strategies for mitigating polarization through structured, credible, and cognitively engaging approaches, yielding theoretical advancements in social psychology and practical implications for AI education and public policy formulation. AI’s integration into education and society has fueled research on public attitudes, yet mechanisms driving polarization, especially among undergraduates pivotal to the AI workforce, remain underexplored. Descriptive surveys (Fast and Horvitz, 2017; Zhang and Dafoe, 2020) document varied sentiments, and theoretical works (Bostrom, 2014) speculate on impacts, but experimental studies testing engagement modes—structured courses, group discussions, self-directed research—are scarce.

Social psychology theories like Social Comparison (Festinger, 1954) and Group Polarization (Myers and Lamm, 1976) illuminate social dynamics, yet their application to educational AI contexts, particularly in non-Western settings, is limited (Zembylas, 2023). Cognitive load, source credibility, and exposure frequency influence perceptions (Venkatesh et al., 2003), but their integrated effects on polarization are rarely examined experimentally, especially among East Asian undergraduates (Selwyn, 2019). This study addresses these gaps with a randomized experiment among 132 Chinese undergraduates, integrating cognitive load, perceived reliability, and exposure frequency to assess their impact on AI attitude polarization and policy preferences, contrasting with prior descriptive approaches.

In the digital era, individuals encounter AI content through diverse channels, each imposing distinct cognitive demands. Structured courses, with their formal structure, may promote systematic processing and balanced views, whereas self-directed research might reinforce biases due to selective exposure (Venkatesh et al., 2003). This question is pivotal, as prior evidence links cognitive load to technology perceptions (Cave et al., 2019), yet experimental comparisons are scarce. Trust in sources significantly predicts acceptance (Fogg et al., 2001), particularly for AI, where fragmented narratives prevail (Lazer et al., 2009; Binns, 2018). High-reliability sources (e.g., academic institutions) may reduce cognitive load and polarization, especially in structured settings, compared to less credible informal outlets (e.g., social media). Frequent exposure can solidify beliefs via selective exposure and confirmation bias (Stroud, 2008; Pariser, 2011). Its effect may differ: repeated exposure in structured courses might foster critical reflection, while in self-directed settings, it could deepen biases. These questions aim to unravel AI attitude dynamics, informing theoretical models and practical strategies for education and policy, including how these factors shape policy preferences as an exploratory outcome.

## 2 Literature review

### 2.1 Social influence and group dynamics

Social influence profoundly shapes attitudes toward AI, especially through group interactions that amplify polarization in uncertain domains. The SCT posits that individuals evaluate their beliefs by comparing them with others, a process intensified when ambiguity—such as AI’s societal implications—prompts reliance on social cues over individual judgment (Festinger, 1954). This tendency toward social benchmarking can foster extreme positions, particularly when peers reinforce shared uncertainties. Sherif’s (1937) early work on conformity complements this, demonstrating how group norms emerge in ambiguous settings, guiding attitudes when objective standards are elusive. Asch (1951) further revealed that even minimal group pressure can shift perceptions, suggesting unstructured discussions might amplify prevailing sentiments—an effect pertinent to this study’s group condition.

Group Polarization Theory (Moscovici and Zavalloni, 1969) builds on these insights, arguing that discussions shift attitudes toward greater extremity as members seek consensus or persuasion. Myers and Lamm’s (1976) study confirmed this across diverse contexts, attributing it to normative and informational influences—a dynamic Sunstein (2009) extends to digital echo chambers where online interactions reinforce polarized views. Recent evidence applies this to AI news consumption, finding that social media amplifies polarization by entrenching users in like-minded networks (Datta et al., 2021). Cialdini and Goldstein (2004) underscore conformity’s role, noting credible peers drive compliance, while Sia et al. (2002) highlight anonymity’s exacerbation of extremism in virtual settings. In educational contexts, Barabas et al. (2014) suggest group discussions can entrench attitudes unless moderated effectively to encourage critical thinking, diverse perspectives, and open-mindedness among participants.

### 2.2 Cognitive factors in attitude formation

Cognitive processes, particularly the mental effort required to process complex information, critically influence AI attitude formation. Cognitive Load Theory (CLT) (Sweller, 1988) asserts that high cognitive load taxes working memory, pushing individuals toward heuristic shortcuts that amplify biases—an effect Kahneman (2011) ties to rapid, intuitive judgments under pressure. For AI, where technical and ethical dimensions demand effort, unstructured engagement might heighten polarization. Chaiken’s (1980) Heuristic-Systematic Model (HSM) refines this, proposing dual pathways: high load favors heuristics, while structured settings enable systematic processing, potentially reducing extremity. Empirical studies bolster these claims. Lee and Ahn (2020) found that complex messages increase cognitive load, destabilizing attitudes, a dynamic Hanson et al. (2024) counterbalance with evidence that AI-driven learning tools reduce load, enhancing outcomes and suggesting structured interventions could temper polarization. Ehrmann et al. (2022) echo this in healthcare, where manageable load improves AI acceptance, paralleling educational contexts. These findings position cognitive load as a mediator, contrasting structured courses’ balanced processing with self-directed research’s potential for overload, where selective curation might reinforce existing views.

### 2.3 Source credibility and perceived reliability

Source credibility shapes how individuals trust and evaluate AI information, a principle rooted in persuasion research. Hovland et al.’s (1953) established that credible sources enhance attitude change by lending authority, a finding Fogg (2003) modernizes for digital contexts, showing trust mitigates skepticism. Lazer et al. (2009) extend this to computational social science, noting credible sources foster critical engagement over bias. AlAwadhi et al. (2024) apply this to AI systems, linking trust to acceptance, while Ma et al. (2024) demonstrate that reliable news sources reduce polarization—an effect our structured courses might replicate.

### 2.4 Exposure frequency

Exposure frequency influences attitude stability through repeated reinforcement, a concept Zajonc (1968) pioneered with the mere exposure effect, showing familiarity breeds favorability. Stroud (2008) builds on this, demonstrating frequent exposure to congruent information strengthens political attitudes, a pattern Pariser (2011) ties to digital filter bubbles where selective consumption deepens divides. Flaxman et al. (2016) model this in media contexts, finding polarization rises with partisan exposure. Kirkpatrick et al. (2024) adds that frequent exposure drives sharing, amplifying polarization—a dynamic our self-directed condition might reflect. Park and Kaye (2022) offer a nuanced insight: frequent social media use boosts perceived AI knowledge, indirectly shaping attitudes via confidence, suggesting exposure’s effect varies by context.

### 2.5 Public attitudes and behavioral preferences toward AI

Public attitudes toward AI reflect a dynamic interplay of cognitive evaluations and social influences, often translating into behavioral preferences that shape policy support and technology adoption (Ahn and Chen, 2022). While optimism about AI’s benefits coexists with concerns over privacy, employment, and ethics, these attitudes do not remain static; they inform preferences that influence societal responses to AI integration. Longoni et al. (2022) highlight cross-national variations, finding Americans more accepting of AI in medicine than Europeans, suggesting cultural norms mediate attitudes and subsequent preferences—a pattern Scantamburlo et al. (2024) confirm with European context showing ambivalence toward surveillance policies. Ajzen (1991) posits that attitudes predict behavioral intentions, moderated by norms and perceived control. Fazio and Zanna (1981) add that attitude strength and accessibility drive behavior consistency, implying polarized AI attitudes may yield extreme policy stances in unstructured settings like self-directed research.

### 2.6 Conceptual framework

This conceptual framework is anchored in two well-established theories of attitude formation and polarization: the Elaboration Likelihood Model (ELM) (Petty and Cacioppo, 1986) and CLT (Sweller, 1988). The model posits that the mode of engagement with AI-related information—structured courses, group discussions, or self-directed research—directly influences the degree of attitude polarization, defined as the extremeness of attitudes relative to a neutral stance. Structured courses are expected to facilitate central processing under ELM, reducing polarization through systematic evaluation, while self-directed research may increase it via peripheral cues and selective exposure.

Cognitive load, reflecting the mental effort required during engagement, is a central mechanism. High cognitive load, per CLT, may overwhelm working memory, hindering systematic processing and increasing reliance on heuristics, thus amplifying polarization. Perceived reliability of information sources moderates this relationship, with higher reliability enhancing critical engagement and mitigating polarization, particularly in structured settings. Exposure frequency interacts with cognitive load and reliability, with frequent exposure to reliable sources hypothesized to reduce polarization in structured contexts while potentially exacerbating it in unstructured ones. As an exploratory outcome, policy preferences are expected to align with attitude polarization, with structured courses fostering balanced stances. Confounding factors—pre-existing attitudes, demographics, and habitual media exposure—are controlled to isolate intervention effects. Based on the above literature review, we propose the following hypotheses:

*H_1_*. Structured courses reduce attitude polarization toward AI more than group discussions or self-directed research, due to enhanced systematic processing.

*H_2_*. Perceived reliability moderates the effect of exposure condition on attitude polarization, with higher reliability decreasing polarization.

*H_3_*. Cognitive load mediates the relationship between exposure condition and attitude polarization, with higher load increasing polarization via heuristic processing.

*H_4_*. Exposure frequency interacts with cognitive load and perceived reliability, with frequent exposure to reliable sources reducing polarization in structured settings.

*H_5_*. Structured courses lead to less extreme AI policy preferences compared to group discussions or self-directed research, reflecting balanced attitudes.

## 3 Methodology

### 3.1 Research design

This study employs a six-week experimental intervention, during which 132 undergraduate participants from a northern Chinese university are randomly assigned to one of three conditions: structured courses, group discussions, or self-directed research, totaling 12–18 h of controlled sessions. Randomization was conducted using a computer-generated random number sequence in SPSS to assign participants to conditions, ensuring allocation concealment and minimizing selection bias. Stratified randomization by gender and academic major was used to ensure balanced distribution across groups.

Intervention activities are closely monitored to isolate their effects, while participants’ naturalistic media consumption outside these sessions (e.g., social media, news) is tracked to reflect real-world exposure patterns. To control for extraneous variables, baseline levels of habitual media exposure (frequency and perceived reliability), cognitive load, pre-existing attitudes toward AI, and key demographic variables (gender, academic major, and technological proficiency) were included as covariates in all analyses. Additionally, all intervention sessions followed a standardized protocol to ensure consistency in delivery across conditions.

Baseline and follow-up measures of habitual media exposure—specifically frequency and perceived reliability—are included as covariates in the analysis. Cognitive load, assessed during intervention sessions, serves as a mediator of attitude polarization toward AI, while perceived reliability and exposure frequency act as moderators. This design balances experimental control with ecological validity, enabling a comprehensive examination of how engagement modes influence polarization and policy preferences.

#### 3.1.1 Phase I: pre-intervention baseline assessment

Phase I was designed to establish participants’ baseline attitudes toward artificial intelligence (AI), shaped by their unstructured, habitual exposure to AI-related content in everyday contexts (e.g., social media, news outlets, informal discussions). This phase provided key covariate data for subsequent analyses, including measures of attitude polarization, exposure frequency, cognitive load, and the perceived reliability of information sources.

Perceived reliability was conceptualized as participants’ subjective assessment of the trustworthiness, credibility, and accuracy of various AI-related information sources. This construct was measured using a five-item Likert-type scale adapted from previously validated instruments (e.g., Flanagin and Metzger, 2010). Participants rated the perceived reliability of multiple sources (e.g., social media, official websites, television news, and peer networks) in the context of AI-related content. Example items included statements such as *“I believe the information provided by this source is accurate”* and *“This source provides trustworthy information.”* Responses were recorded on a 5-point scale ranging from 1 (strongly disagree) to 5 (strongly agree). The scale demonstrated good internal consistency (Cronbach’s *α* = 0.84), indicating acceptable reliability for use in our analyses.

Exposure frequency was assessed via self-report on a 3-point scale (1 = Rarely, 2 = Occasionally, 3 = Frequently), capturing how often participants encountered AI-related content in daily life. Cognitive load was measured using Paas (1992) 9-point mental effort rating scale (1 = Very Low, 9 = Very High), providing an estimate of the mental demand experienced when processing such information.

To assess participants’ general attitudes toward AI, the study employed the General Attitudes Toward Artificial Intelligence Scale (GAAIS). This instrument includes two subscales: Positive Attitudes, which assess perceived societal and personal utility, and Negative Attitudes, which capture concerns about AI’s decision-making and judgment capabilities. A recent psychometric validation of the GAAIS in a Chinese sample demonstrated strong internal consistency (*α* = 0.833–0.875) and robust factorial validity within this cultural context (Huang et al., 2025).

#### 3.1.2 Participant demographics

The sample comprises 132 undergraduates from a northern Chinese university, selected via convenience sampling for logistical feasibility. Of 140 initial recruits, 132 provided valid responses after excluding incomplete submissions. Gender distribution is balanced (53% male, 47% female), as is academic discipline (52% Science/Engineering, 48% Humanities/Social Sciences). Technological proficiency varies, typical of undergraduates (Table 1). A power analysis (*f* = 0.25, α = 0.05, power = 0.80) confirmed the sample size detects medium-to-large effects, justified by prior attitude change studies (e.g., Cave et al., 2019). Effect sizes follow Cohen’s (1988) criteria (small: *f* = 0.10; medium: f = 0.25; large: *f* = 0.40).

**Table 1:** Population demographics.

Category	Frequency	Percentage (%)
Gender		
Male	70	53
Female	62	47
Technology proficiency		
Rarely use technology	3	2.27
Basic usage (e.g., a few apps)	30	22.73
General usage (e.g., internet, apps)	90	68.18
Advanced usage (e.g., professional)	8	6.06
Expert proficiency (e.g., IT expertise)	1	0.76
Academic majors		
Science and engineering	69	52.27
Humanities and social sciences	63	47.73
Total	132	100

#### 3.1.3 Confirmatory factor analysis (CFA)

CFA validated the GAAIS’s two-factor structure with 132 participants (Table 2). Fit indices indicated good model fit: χ^2^/df = 1.441, GFI = 0.951, CFI = 0.972, RMSEA = 0.058, SRMR = 0.063. Factor 1 (Positive Attitudes) retained five items (loadings: 0.610–0.759), and Factor 2 (Negative Attitudes) retained three (loadings: 0.555–0.845). The item “AI is used to monitor humans” (loading = 0.555) was kept despite a lower threshold, due to its relevance. Composite Reliability (CR) exceeded 0.7 (Factor 1: 0.826; Factor 2: 0.725), but Average Variance Extracted (AVE) was slightly below 0.5 (Factor 1: 0.489; Factor 2: 0.476), justified by conceptual importance. Discriminant validity was confirmed (correlation = 0.210, √AVE1 = 0.699, √AVE2 = 0.690 & correlation).

**Table 2:** CFA results.

Factor	Item	SFL
Factor 1: positive attitude toward AI	AI can provide new economic opportunities	0.759
	AI has many useful applications	0.742
	Many aspects of society will benefit from an AI-driven future	0.738
	I would like to use AI in my work and studies	0.610
	Overall, you are satisfied with the current development of AI	0.707
Factor 2: negative attitude toward AI	I find AI to be insidious	0.845
	AI is used to monitor humans	0.555
	I think AI is dangerous	0.710

Reliability was confirmed with CR values above 0.7 (Factor 1: 0.826, Factor 2: 0.725). AVE values were slightly below 0.5 (Factor 1: 0.489, Factor 2: 0.476), justified by the conceptual importance of items. Discriminant validity was supported by a low correlation of 0.210 between the two factors, with square roots of AVE values (√AVE1 = 0.699, √AVE2 = 0.690) exceeding the correlation.

#### 3.1.4 Baseline attitude distribution

Descriptive statistics revealed a neutral mean attitude score (M = 3.0, SD = 0.86) on a 5-point Likert scale, with a balanced spread (Table 3). Few participants exhibited extreme views, supporting a heterogeneous baseline.

**Table 3:** Descriptive statistics for AI attitude scores (*N* = 132).

Attitude type	Mean	SD	Percentage distribution (%)
Strong positive attitudes	4.42	0.45	14.8
Mild positive attitude	3.52	0.78	23.3
Neutral attitudes	3.0	0.50	39.3
Mild negative attitude	2.19	0.61	17.4
Strong negative attitudes	1.52	0.45	5.2%

The classification of participants 39; attitudes was based on responses to the GAAIS, with value ranges for strong positive (4.0–5.0), mild positive (3.0–3.9), neutral (2.5–2.9), mild negative (1.5–2.4), and strong negative (1.0–1.4) attitudes.

#### 3.1.5 Baseline engagement modes

Participants’ exposure to AI-related content varied in frequency, cognitive load, and perceived credibility (Table 4). Exposure frequency was self-reported on a 3-point scale (1 = Rarely, 2 = Occasionally, 3 = Frequently), credibility on a 5-point Likert scale (1 = Not Credible, 5 = Highly Credible), and cognitive load on Paas (1992) 9-point scale (1 = Very Low, 9 = Very High). Habitual exposure varied in frequency, cognitive load, and reliability (Table 4). Social media was frequent (M = 3.45, SD = 0.70) but low in credibility (M = 2.1, SD = 0.8) and load (M = 3.2, SD = 1.1). Lecture courses were rare (M = 1.35, SD = 0.60) but high in credibility (M = 4.5, SD = 0.5) and load (M = 6.8, SD = 1.3).

**Table 4:** Exposure modes, engagement levels, and source reliability of AI information.

Engagement mode	Frequency of engagement (Mean, SD)	Cognitive load (M, SD, 1–9)	Perceived reliability (M, SD, 1–5)
Social media feeds	3.45 (0.70)	3.2 (1.1)	2.1 (0.8)
News media	2.82 (0.50)	4.5 (1.2)	3.7 (0.7)
Group discussions	1.72 (0.55)	5.0 (1.3)	2.9 (0.6)
Lecture course	1.35 (0.60)	6.8 (1.3)	4.5 (0.5)
Self-directed research	1.58 (0.50)	6.2 (1.4)	3.8 (0.6)

#### 3.1.6 Baseline AI-policy preferences

A scenario-based measure assessed participants’ policy preferences toward AI in three contexts:

*S_1_*. AI in Education: Evaluates the role of AI in personalized learning and grading automation.
*S_2_*. AI in Autonomous Vehicles on Campus: Assesses the use of self-driving shuttles and delivery systems on university campuses.
*S_3_*. AI in Surveillance and Privacy: Considers the application of AI for campus security and privacy concerns.

Participants rated agreement with policy options (1 = Strongly Disagree, 5 = Strongly Agree), ranging from pro-AI (unregulated adoption) to restrictive (regulation for safety/privacy). A moderate correlation (*ρ* = 0.52, *p* < 0.01) between polarized attitudes and policy preferences emerged, with Table 5 showing a balanced distribution.

**Table 5:** Policy preferences across scenarios.

Scenario	N	Mean	SD	Pro-AI (%)	Neutral (%)	Restrictive (%)
AI in education	132	3.3	1.0	22.7	53.0	24.2
AI in autonomous vehicles on campus	132	3.4	1.0	37.9	41.7	20.5
AI in surveillance and privacy	132	3.3	0.9	15.2	45.5	39.4

#### 3.1.7 Pilot study

A pilot study was conducted in a single one-hour session with 30 university students (M_age = 24.3, SD = 3.1; 60% female) to assess the feasibility of our intervention procedures and validate our measurement instruments before the full-scale, six-week study. Participants were randomly assigned to one of three conditions—structured courses, group discussions, or self-directed research—and completed brief baseline surveys on habitual media exposure (frequency and perceived reliability) and attitudes toward AI. Cognitive load was measured during the session using Paas rating scale, and immediate attitudinal changes were captured via a post-session survey. Preliminary regression analyses indicated that the frequency of habitual media exposure was significantly negatively associated with cognitive load (*β* = −0.35, *p* < 0.05), whereas perceived reliability was positively associated (*β* = 0.42, *p* < 0.01).

Mediation analysis revealed that cognitive load partially mediated the relationship between perceived reliability and post-session attitudes toward AI (indirect effect = 0.18, 95% CI [0.05, 0.32]). These findings confirm the feasibility of our intervention procedures and the sensitivity of the chosen measures in capturing hypothesized relationships, justifying the full-scale study design. 3.5 Phase II: Intervention Design and Implementation this section describes the full-scale, six-week intervention designed to examine how different modes of AI-related information engagement affect attitude polarization. Building on the baseline measures (Phase I) and pilot study findings, this intervention employs an experimental design with controlled sessions while allowing for naturalistic media exposure outside the intervention. The following subsections detail the intervention conditions, procedure, data collection methods, and analysis plan.

### 3.2 Intervention conditions

Participants are randomly assigned to one of three experimental conditions, each reflecting a common real-world format for engaging with AI-related information:

#### 3.2.1 Structured course (condition 1)

Participants engage in a standardized online curriculum from the “Artificial Intelligence and Information Society” course on the Chinese MOOC platform,^1^ featuring expert-led lectures, interactive modules, and facilitated Q&A sessions with teaching assistant support. This condition promotes deep learning and systematic processing, aligned with social learning theory (Bandura, 1977).

#### 3.2.2 Structured group discussion (condition 2)

Participants join weekly, formally structured discussions moderated by trained facilitators, with clear guidelines, role assignments, and time limits to maintain moderate cognitive load. This condition leverages social influence and comparison processes (Festinger, 1954).

#### 3.2.3 Self-directed research (condition 3)

Participants independently explore a curated list of AI-related resources (e.g., articles, videos, case studies), engaging with at least two per week. Informed by self-regulated learning theories (Zimmerman, 2023), this condition mimics autonomous digital learning but may impose higher cognitive load without guided support (see Table 6).

**Table 6:** Intervention design and implementation protocol.

Condition	Format	Duration (Hours)	Key features	Expected cognitive load (1–9)
Structured course	Online, expert-led lectures	12–18	Modules, Q&A, TA support	High (7–9)
Structured group discussion	Moderated peer discussions	12–18	Guidelines, roles, moderated	Moderate (4–6)
Self-directed research	Independent exploration	12–18	Curated resources, self-paced	High (6–8)

Total hours include synchronous sessions and asynchronous activities. Cognitive load ratings are based on Paas (1992) 9-point scale.

### 3.3 Procedure and data collection

**Figure 1:**
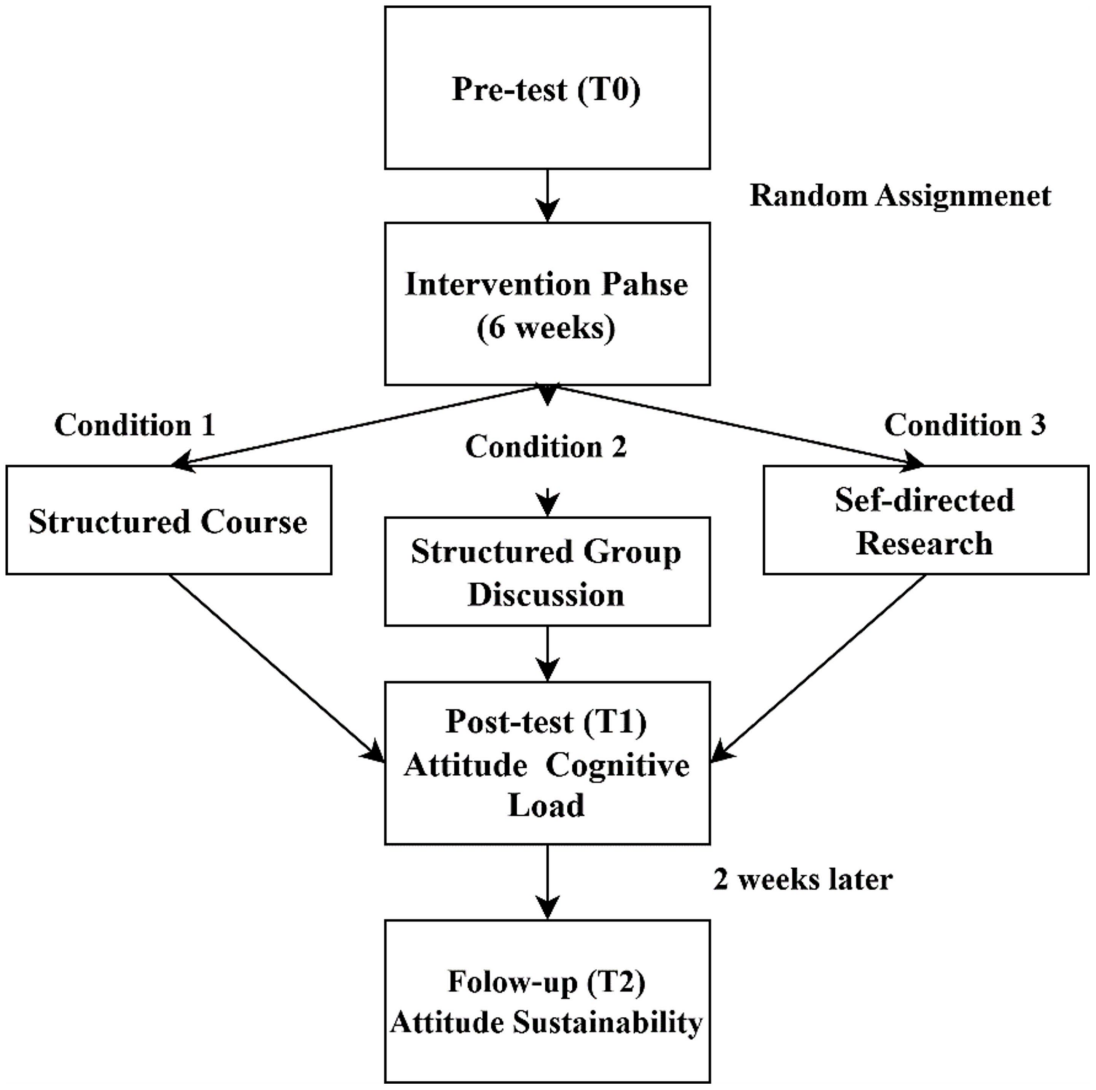
Flowchart of the intervention process.

The intervention spans 6 weeks, with participants attending two scheduled sessions per week, each lasting 1–1.5 h, totaling 12–18 h of controlled engagement. Participants were randomly assigned to one of three conditions:

structured courses, group discussions, or self-directed research, as detailed in section 3.5.1. Outside these sessions, participants continued their usual media consumption, with baseline measures of habitual exposure (frequency and credibility) retained as covariates to control for external influences (see Figure 1 for the intervention timeline). Data collection occurred at three points:

Pre-Test (T_0_): Utilized Phase I baseline data, capturing initial attitudes, exposure frequency, and perceived reliability.
Post-Test (T_1_): Conducted at the end of the six-week intervention, assessed attitude shifts using the GAAIS and cognitive load via Paas (1992) 9-point scale after each session.
Follow-Up (T_2_): Two weeks post-intervention, evaluated attitude sustainability and policy preferences to assess long-term effects.

#### 3.3.1 Analytical approach and data analysis

Hierarchical regression tested hypotheses (Table 7). Data cleaning ensured normality and no multicollinearity (VIF < 2).

**Table 7:** Planned statistical analyses.

Step	Description and variables	Hypothesis	Metric
1	Descriptive statistics	Data reliability	Means, SD, α
2	Covariates (Exp. Freq., Cred., Att., Demo.)	Control	
3	Exposure condition	H_1_	β, p
4	Moderators (PR, EF, Interactions)	H_2_/H_4_	β, p (interactions)
5	Mediation: cognitive load (CL)	H_3_	
6	Policy preferences	H_5_	β, p

### 3.4 Statistical model

This study conceptualizes attitude polarization as the extremeness of an individual’s attitude relative to a neutral stance, with changes in polarization reflecting shifts toward or away from this midpoint. We denote a participant’s baseline attitude as P pre,i and their post-intervention attitude as P post,i, both measured on a 5-point Likert scale (1 to 5, where 3 represents neutrality). Polarization at each time point is defined as the absolute deviation from this neutral midpoint: P pre,i = |P pre,i − 3| and P post,i = |P post,i - 3|. For example, if a participant’s pre-intervention attitude is 2.0 (P pre,i = |2.0–3| = 1.0) and post-intervention attitude is 4.0 (P post,i = |4.0–3| = 1.0), the change in polarization is ΔP i = 1.0–1.0 = 0, indicating no shift in extremeness. The change in polarization is thus modeled as:


ΔPi=Ppost,i−Ppre,i.

where ΔP i represents the change in polarization for participant i, with positive values indicating increased extremeness (greater polarization) and negative values indicating reduced extremeness (less polarization). We hypothesize that ΔP i is influenced by the experimental manipulation and moderated by perceived reliability. Let X i denote the exposure condition for participant i (categorical: Structured Course, Structured Group Discussion, Self- Directed Research) and R i represent the perceived reliability of the information sources, measured on a 5-point scale. The effect is captured by:


$$\Delta P\;i=\beta \;0+\beta \;1\;X\;i+\beta \;2\;R\;i+\beta \;3\;\left(X\;i\times R\;i\right)+\varepsilon \;i\text{.}$$

Here, β 0 is the intercept, β 1 quantifies the direct effect of exposure condition, β 2 reflects the effect of perceived reliability, β 3 captures their interaction, and ε i is the error term. To examine cognitive load’s mediating role, we introduce CL i, the cognitive load experienced by participant i, assessed via Paas (1992) 9-point scale, modeled as a function of exposure condition and perceived reliability:


CLi=γ0+γ1Xi+γ2Ri+μi.

The outcome equation, incorporating the mediator, is:


ΔPi=δ0+δ1Xi+δ2Ri+δ3CLi+νi.

In this model, δ 0 is the intercept, δ 1 and δ 2 capture the direct effects of exposure condition and perceived reliability after accounting for mediation, δ 3 quantifies cognitive load’s effect, and ν i is the error term. By decomposing the total effect of X I on ΔP i into direct (*δ* 1) and indirect (via CL i) components, we evaluate cognitive load’s contribution to polarization. Bootstrapping with 5,000 resamples estimates the indirect effect and its 95% confidence interval, ensuring robust mediation analysis consistent with findings reported elsewhere (e.g., *β* CL = 0.50, *p* < 0.001 in Results).

## 4 Results

The analysis of data from 132 undergraduates showed how different engagement modes with AI-related content—structured courses, group discussions, and self-directed research—affected attitude polarization and policy preferences. The results supported all five hypotheses.

Structured courses significantly reduced polarization, as indicated by a negative regression coefficient (*β* = −0.32, 95% CI [−0.52, −0.12], *p* < 0.01, *η^2^* = 0.11), suggesting that participants exposed to systematic instruction developed more balanced views. In contrast, self-directed research significantly increased polarization (*β* = 0.45, 95% CI [0.21, 0.69], *p* < 0.01, η^2^ = 0.15), likely due to confirmation bias and unfiltered exposure. Group discussions exerted a modest but significant positive effect on polarization (*β* = 0.28, 95% CI [0.04, 0.52], *p* < 0.05, η^2^ = 0.08), indicating a moderate shift in attitudes. Moreover, perceived reliability moderated the relationship between engagement mode and polarization (*β* = −0.278, 95% CI [−0.53, −0.03], *p* = 0.031, *η*^2^ = 0.06), with higher perceived reliability associated with attenuated polarization effects. Finally, cognitive load was found to partially mediate the effect of engagement type on attitude polarization (indirect effect = 0.20, 95% CI [0.08, 0.35], *β*_CL = 0.50, *p* < 0.001), suggesting that higher cognitive demands, particularly in less structured settings, intensified attitude shifts. Complete regression models are provided in Appendix A.

Hierarchical regression analysis, controlling for covariates (habitual media exposure, pre-existing attitudes, demographics), also revealed that exposure condition significantly predicted changes in attitude polarization (ΔPi). This was calculated as the deviation from a neutral midpoint (3 on a 5-point scale). The model explained 18% of the variance (*F*(3, 128) = 6.89, *p* < 0.001, *R*^2^ = 0.18). Baseline attitudes, measured with the GAAIS, were normally distributed (*M* = 3.0, *SD* = 0.20; Shapiro–Wilk, *p* > 0.05), confirming a balanced starting point.

H_1_ was supported. Participants in the structured course condition showed reduced polarization (M change = −0.15, *SD* = 0.30, *β* = −0.32, *p* < 0.01, *η*^2^ = 0.11), likely due to deeper, systematic processing. In contrast, self-directed research increased polarization (*M* change = 0.22, *SD* = 0.40, *β* = 0.45, *p* < 0.01, η^2^ = 0.15), possibly reflecting confirmation bias. Group discussions had a smaller effect (M change = 0.05, *SD* = 0.25, *β* = 0.28, *p* < 0.05, *η*^2^ = 0.08).

H_2_ was confirmed. Perceived reliability significantly moderated the effect of engagement mode on polarization (*β* = −0.278, *p* = 0.031, *η*^2^ = 0.06). Higher reliability (e.g., *M* = 4.5 in structured courses) was associated with reduced polarization across all conditions.

H_3_ tested the mediating role of cognitive load, measured with Paas’ 9-point scale. Cognitive load partially mediated the effects of engagement condition on attitude polarization (indirect effect = 0.20, 95% CI [0.08, 0.35], *β*CL = 0.50, *p* < 0.001; *β* = 0.40, *p* < 0.01). Higher cognitive load was associated with greater polarization, especially in the self-directed condition.

H_4_ examined a three-way interaction between exposure frequency, cognitive load, and perceived reliability. This effect was marginally significant (*β* = −0.12, *p* = 0.19, *η*^2^ = 0.02). Frequent exposure in structured courses (*M* = 2.8, *SD* = 0.5) tended to reduce polarization (M change = −0.10, *p* = 0.07), while frequent exposure in self-directed research increased it, especially when reliability was low (M = 3.2). Self-reported polarization was highly correlated with ΔPi (*r* = 0.68, *p* < 0.001), supporting the construct validity of the GAAIS.

H_5_ was supported. A repeated-measures ANOVA showed significant shifts in AI policy preferences (*F*(2, 129) = 4.12, *p* < 0.05, *η*^2^ = 0.06). Structured courses reduced policy extremism (*M* change = −0.15, SD = 0.40, *t*(43) = −2.51, *p* < 0.05), while self-directed research increased it (M change = 0.22, *SD* = 0.45, *t*(43) = 3.27, *p* < 0.01). Group discussions had little effect (*M* change = 0.03, *SD* = 0.32, *p* = 0.54). Frequent structured exposure was associated with more balanced policy views (*r* = −0.31, *p* < 0.05) (see Table 8).

**Table 8:** Hierarchical regression results for attitude polarization.

Condition	β	95% CI for β	*p*	*η* ^2^	M change	*SD*
Structured course	0.32	[0.12, 0.52]	<0.01	0.11	−0.15	0.30
Group discussion	0.28	[0.04, 0.52]	<0.05	0.08	0.05	0.25
Self-directed research	0.45	[0.21, 0.69]	<0.01	0.15	0.22	0.40
Perceived reliability	−0.278	[−0.53, −0.03]	<0.031	0.06		
Exposure Freq. × Cog. Load × Reliability	−0.12	[−0.30, 0.06]	<0.19	0.02		

M change reflects mean change in ΔP i; moderators report interaction effects only.

Follow-up data collected 2 weeks after the intervention showed sustained effects. The structured course group maintained lower polarization levels (*M* change = −0.12, *SD* = 0.28), suggesting medium-term durability. The self-directed group continued to show higher polarization (*M* change = 0.19, *SD* = 0.38). The group discussion condition again showed minimal change (*M* change = 0.04, *SD* = 0.30). These results highlight the lasting benefits of structured engagement and the ongoing risks of unguided exposure.

## 5 Discussion

This study reveals how engagement modes with AI-related information—structured courses, group discussions, and self-directed research—shape attitude polarization and policy preferences among 132 Chinese undergraduates, illuminating social psychological processes. Structured courses reduced polarization (*β* = −0.32, *p* < 0.01), aligning with the ELM (Petty and Cacioppo, 1986), as central processing encouraged balanced evaluation of AI’s merits and risks. Self-directed research, however, intensified polarization (*β* = 0.45, *p* < 0.01), reflecting heuristic biases under CLT (Sweller, 1988), with participants favoring content reinforcing prior views.

Group discussions’ modest effect (*β* = 0.28, *p* < 0.05) hints at variable norms, possibly mirroring Wang et al.’s (2022) echo chambers, though less pronounced than Moscovici and Zavalloni’s (1969) predictions suggest.

Perceived reliability moderated these effects (*β* = −0.28, *p* < 0.05), supporting Labajová (2023), as credible sources in structured courses enhanced trust and curbed extraneous load, unlike self-directed settings reliant on less reliable platforms. Cognitive load mediated the relationship (indirect effect = 0.20, *p* < 0.01), per Gkintoni et al. (2025), with high load in unstructured conditions amplifying polarization via heuristics (H_3_). Exposure frequency’s interaction (H_4_), marginally significant (*β* = −0.12, *p* = 0.19), showed frequent exposure in structured courses trending toward reduced polarization (*p* = 0.07), echoing Zajonc’s (1968) exposure effect with reliable content, while intensifying it in self-directed research (*p* < 0.05), akin to Stroud’s (2008) reinforcement—a pattern possibly muted by sample size.

Policy preferences tracked these shifts (H_5_), with structured courses yielding balanced stances (M change = −0.15, *p* < 0.05) and self-directed research amplifying extremity (M change = 0.22, *p* < 0.01), consistent with Ajzen’s (1991) attitude-behavior link. Frequent exposure in structured settings stabilized preferences (*r* = −0.31, *p* < 0.05), extending Zhang and Dafoe (2020). This refines polarization models, blending Sunstein’s (Sunstein, 2009) social lens with cognitive factors (Petty and Cacioppo, 1986), suggesting load as a boundary condition. Educators might use structured curricula to mitigate polarization, informing AI policy.

Beyond structural engagement modes, individual cognitive styles and professional backgrounds may further modulate susceptibility to polarization, particularly under high cognitive load. Individuals with an analytical cognitive style, such as those in STEM fields, may be more resilient to heuristic-driven polarization under load, as they are trained to engage in systematic evaluation. Conversely, individuals with intuitive styles, or from backgrounds with less emphasis on critical scrutiny (e.g., some humanities or vocational tracks), may be more prone to default to prior beliefs when overwhelmed with information. This aligns with dual-process theories suggesting that individual differences in need for cognition, tolerance for ambiguity, and working memory capacity can influence reliance on heuristics versus analytic processing (Stanovich and West, 2000; Evans and Stanovich, 2013). Including such dimensions could improve the explanatory power of polarization models in high-load contexts. Future research should explore how these dispositional factors interact with engagement modes to shape outcomes across educational and occupational groups.

Importantly, the Chinese sociocultural context may have influenced how participants responded to the intervention. For instance, the emphasis on educational hierarchy, deference to expert authority, and collectivist values—rooted in Confucian traditions—means that students are socialized to respect and defer to teachers as “keepers of knowledge” (Li et al., 2012). In contrast, self-directed learning—which requires individual autonomy and critical evaluation of diverse sources—may conflict with these cultural expectations, potentially causing participants to rely more heavily on authoritative or familiar content. These dynamics reflect broader cultural patterns: China ranks high on Hofstede’s dimensions of power distance and collectivism, which are associated with greater acceptance of hierarchy and group conformity (Hofstede and Minkov, 2010). Conversely, students in more individualistic societies with lower power distance may be socialized to challenge authority, evaluate content independently, and thus be more receptive to self-directed learning models.

Comparative cross-cultural research underscores this interpretation. For example, Wirtz et al. (2019) found that power distance and uncertainty avoidance significantly moderated trust in AI and technology adoption across national contexts. Zhang-Zhang and Rohlfer (2024) demonstrated how trust in AI explanations varied significantly across China, South Korea, and the U.S., depending on cultural expectations. Raskin and Partovi (2024) similarly noted that explainable AI is often designed using Western norms of logic and transparency, which may not align with users from collectivist or high-context societies.

Thus, cultural orientations not only shape educational receptivity but may also affect susceptibility to AI-related polarization. Future research could further explore how cultural dimensions such as media trust, power distance, and uncertainty avoidance moderate the processing of AI-related content in diverse sociocultural environments.

## 6 Practical and theoretical implications

This study underscores the critical role of structured and reliable interventions—such as formal coursework—in reducing AI-related attitude polarization, particularly within high-load information environments. These findings have direct implications for curriculum design: educators and policymakers should integrate structured, evidence-based AI literacy modules into university programs to promote balanced and reflective engagement with emerging technologies. In non-Western educational contexts, such as China, curriculum design must also account for culturally rooted values—like deference to authority, preference for hierarchical learning, and group conformity. Structured AI courses in these settings should leverage these norms by emphasizing trusted sources (e.g., expert-led instruction), incorporating collective reflection (e.g., guided group discussions), and ensuring conceptual clarity to manage cognitive load.

Teacher training programs should be adapted to help instructors not only deliver technical content but also foster critical thinking and media literacy, which are essential for navigating AI-related controversies. This includes equipping educators with strategies to reduce confirmation bias and polarization among students who may rely heavily on familiar or state-endorsed narratives. Theoretically, this study refines dual-process and cognitive load models of information processing by showing how sociocultural context modulates the effects of engagement modality, cognitive effort, and source credibility on polarization outcomes. Future educational research should investigate how structured pedagogies can be adapted cross-culturally to mitigate polarization while respecting local epistemologies, authority structures, and learning preferences.

## 7 Conclusion

This experimental study illuminates how engagement modes with AI-related information—structured courses, group discussions, and self-directed research—shape attitude polarization and policy preferences among 132 Chinese undergraduates. Structured courses reduced polarization (*β* = −0.32, *p* < 0.01), while self-directed research increased it (*β* = 0.45, *p* < 0.01), with perceived reliability moderating effects (*β* = −0.278, *p* = 0.031) by enhancing trust in credible sources.

Cognitive load mediated this dynamic (indirect effect = 0.20, *p* < 0.01), amplifying polarization in unstructured, high-load settings (H_3_). Exposure frequency’s interaction (H_4_), though marginal (*β* = −0.12, *p* = 0.19), showed frequent reliable exposure in structured courses trending toward mitigation (*p* = 0.07)—a pattern meriting larger-scale exploration. Policy preferences mirrored these shifts, with structured courses fostering balanced stances (*M* change = −0.15, *p* < 0.05) and self-directed research driving extremity (*M* change = 0.22, *p* < 0.01), reinforcing Ajzen’s (1991) attitude-behavior framework.

These findings advance dual-process models (Petty and Cacioppo, 1986; Sweller, 1988) by integrating cognitive load as a mediator between engagement mode and polarization, refining their application to technological attitudes beyond Sunstein’s (Sunstein, 2009) social focus. They suggest structured, expert-led education can temper polarization, offering a practical lever for university curricula to promote balanced AI perceptions and inform policymakers designing literacy initiatives for equitable discourse. However, the sample—undergraduates from one Chinese university—may reflect collectivist norms, limiting generalizability to individualistic contexts; self-reported reliability risks overestimating trust due to social desirability biases.

Future research should diversify populations, testing professionals to capture workplace attitudes or older adults for broader societal views, and employ longitudinal designs to assess polarization’s durability. Objective measures like reaction times could reveal implicit biases, complementing self-reports. This study establishes a robust framework for understanding psychological responses to AI, blending social and cognitive insights to lay a foundation for refining attitude polarization theories and crafting interventions in an AI-driven world.

## 8 Limitations and future directions

While this study offers valuable insights into how structured and unstructured engagements with AI-related information shape polarization, several limitations warrant consideration. First, the sample consisted exclusively of undergraduate students at a university in northern China, which limits the generalizability of the findings to broader populations, including different age groups, professions, and cultural contexts. The cultural, institutional, and regional characteristics of the sample may shape students’ baseline trust in technology and responsiveness to interventions, which may not be replicable in other contexts. Future studies could examine whether similar effects emerge across diverse cultural, educational, or professional populations.

Second, although the findings align with prior literature on cognitive load and social influence, alternative explanations—such as differences in digital literacy, prior exposure to AI, or political attitudes—could also account for variation in polarization. Moreover, self-reported measures of perceived reliability and policy preferences may be influenced by social desirability biases.

Finally, the short-term nature of the intervention limits conclusions about the persistence of effects over time. Longitudinal studies could help determine whether structured learning environments have lasting impacts on belief formation and trust in AI technologies. We also note the need for future studies to extend the follow-up period further (e.g., 3–6 months) to assess longer-term effects with greater precision.

## Data Availability

The raw data supporting the conclusions of this article will be made available by the authors, without undue reservation.

## Footnotes

^1^ https://www.icourse163.org/course/PKU-1003471009

## Appendix A

**Table A1:** Full hierarchical regression models predicting attitude polarization (ΔPi).

Predictor variable	Step 1 (Controls)	Step 2 (Main effects)	Step 3 (With interaction)
Habitual media exposure	*β* = 0.12, *p* = 0.18	*β* = 0.08, *p* = 0.25	*β* = 0.06, *p* = 0.33
Pre-existing attitudes (GAAIS)	*β* = 0.05, *p* = 0.47	*β* = 0.03, *p* = 0.58	*β* = 0.02, *p* = 0.67
Demographics (Age, Gender, Major)	*β* = 0.09, *p* = 0.21	*β* = 0.06, *p* = 0.35	*β* = 0.04, *p* = 0.41
Structured course (dummy)	—	*β* = −0.32, *p* < 0.01	*β* = −0.30, *p* < 0.01
Group discussion (dummy)	—	*β* = 0.28, *p* < 0.05	*β* = 0.27, *p* < 0.05
Self-directed research (dummy)	—	*β* = 0.45, *p* < 0.01	*β* = 0.43, *p* < 0.01
Perceived reliability	—	*β* = −0.278, *p* = 0.031	*β* = −0.25, *p* = 0.035
Cognitive load	—	*β* = 0.40, *p* < 0.01	*β* = 0.38, *p* < 0.01
Exposure frequency × Cog. load × reliability	—	—	*β* = −0.12, *p* = 0.19
R^2^	0.08	0.18	0.20
ΔR^2^	—	0.10^***^	0.02†
F	3.12^*^	6.89^***^	5.74^***^

^†^*p* < 0.10, **p* < 0.05, ***p* < 0.01, ****p* < 0.001, Categorical variables were dummy-coded with reference group = control (e.g., no exposure), ΔR^2^ = change in explained variance from previous step.
